# Role of Oral and Gut Microbiota in Dietary Nitrate Metabolism and Its Impact on Sports Performance

**DOI:** 10.3390/nu12123611

**Published:** 2020-11-24

**Authors:** Rocío González-Soltero, María Bailén, Beatriz de Lucas, Maria Isabel Ramírez-Goercke, Helios Pareja-Galeano, Mar Larrosa

**Affiliations:** 1 Faculty of Biomedical and Health Sciences, Universidad Europea de Madrid, Villaviciosa de Odón, 28670 Madrid, Spain; maria.bailen@universidadeuropea.es (M.B.); mariaisabel.ramirez@universidadeuropea.es (M.I.R.-G.); 2 Faculty of Sports Sciences, Universidad Europea de Madrid, Villaviciosa de Odón, 28670 Madrid, Spain; beatriz.delucas@universidadeuropea.com (B.d.L.); helios.pareja@universidadeuropea.es (H.P.-G.); mar.larrosa@universidadeuropea.com (M.L.)

**Keywords:** nitrite metabolism, nitric oxide, microbiota-muscle axis, *Veillonella*, nitrate supplementation, sports performance

## Abstract

Nitrate supplementation is an effective, evidence-based dietary strategy for enhancing sports performance. The effects of dietary nitrate seem to be mediated by the ability of oral bacteria to reduce nitrate to nitrite, thus increasing the levels of nitrite in circulation that may be further reduced to nitric oxide in the body. The gut microbiota has been recently implicated in sports performance by improving muscle function through the supply of certain metabolites. In this line, skeletal muscle can also serve as a reservoir of nitrate. Here we review the bacteria of the oral cavity involved in the reduction of nitrate to nitrite and the possible changes induced by nitrite and their effect on gastrointestinal balance and gut microbiota homeostasis. The potential role of gut bacteria in the reduction of nitrate to nitrite and as a supplier of the signaling molecule nitric oxide to the blood circulation and muscles has not been explored in any great detail.

## 1. Introduction

Recent studies describing the improvements in skeletal muscle function induced by nitrate supplementation have provided some evidence implicating the oral and gut microbiota as mediators in the underlying mechanisms. Yet, very little is known about how nitrate intake can enhance sports/exercise performance, and further investigation is needed to minimize the potential health risks associated with the dietary consumption of nitrate-containing foods. The efficacy of acute nitrate supplementation seems to be dependent on many factors such as age, health, diet, and fitness/training status, and it has the overall ability to rapidly influence vascular tone and peripheral tissue oxygenation [1,2,3]. The effect of longer-term nitrate supplementation for sports performance is, however, unclear. With regards to its impact on indices of exercise performance in healthy volunteers, the literature seems consistent in reporting that 2–6 days (or up to 15 days) of nitrate supplementation can increase sports performance [3].

Nitrate is naturally present in vegetables, particularly leafy vegetables, and both nitrate and nitrite are used as additives in processed meats. Historically, exogenous nitrate and nitrate have been considered as environmental pollutants and a potential source of health problems, as they can lead to the formation of probable carcinogenic N-nitrosamines [4,5], which are common in nitrite/nitrate-treated meat and poultry products. Accordingly, several restrictions have been placed upon the levels of acceptable nitrate, particularly in processed foods and cured meat [6]. In contrast to exogenous nitrate, many studies (reviewed in [7]) point to endogenous nitrate as an active component in vegetables that contributes to the beneficial health effects of this food group including protection against cardiovascular disease and type II diabetes. Regarding the intake of dietary nitrate/nitrate, both the former Scientific Committee on Food of the European Commission and the Joint FAO/WHO Expert Committee on Food Additives have established that the acceptable daily intake is 3.7 mg/kg bw/day [8]. The expert consensus is that nitrate supplementation with vegetable products such as beetroot juice is likely not harmful. Dietary nitrate is a precursor of the signaling molecule nitric oxide (NO), and has been recently identified both as a therapeutic agent [9] and an ergogenic aid [10]. Indeed, according to the Sports Supplement Framework developed by the Australian Sports Institute, which categorizes supplements into four categories based on scientific evidence (A, B, C, and D), nitrate has been considered as category A (a performance supplement) [11]. This is supported by other studies from the International Olympic Committee [10] and our own laboratory [12]. As mentioned earlier, the biological mechanisms by which dietary nitrate exerts its ergogenic effects is through direct vasodilatation, which promotes muscle blood flow, increasing oxygen consumption efficiency [13,14] and decreasing blood pressure [15,16,17,18]. It is known that dietary nitrate reduces the oxygen cost during physical activity and improves exercise tolerance [19,20,21]. A proposed mechanism to explain this effect is the improvement in mitochondrial efficiency. Indeed, nitrate affects basal mitochondrial function by enhancing oxidative phosphorylation efficiency (amount of oxygen consumed per ATP produced, P/O ratio), which correlates with the reduction of the oxygen cost during exercise [14].

A recent systematic review [19] suggested that nitrate supplementation (mainly tested in the form of beetroot juice) improves cardiorespiratory endurance in athletes by increasing skeletal muscle efficiency in oxygen uptake. Nitrate supplementation also enhances performance at various distances, increases the time to exhaustion at submaximal intensities, and may also improve cardiorespiratory performance at anaerobic threshold intensities and maximum oxygen uptake (VO_2max_). The ergogenic effects of nitrate have been demonstrated not only in endurance and submaximal exercises [13,22,23], but also in resistance-based exercises [12].

It has been extensively discussed that nitrate plays an important effect after exercise, being necessary to induce the vascular response in the first period of recovery by lowering blood pressure and stimulating greater skeletal muscle oxygenation [24]. Of note, the biological effects of dietary nitrate can be suppressed by the use of oral anti-bacterial mouthwash, which has been shown to reduce plasma nitrite levels [25,26,27,28] and to increase the risk of hypertension [29].

Several studies have hypothesized an important role for the oral microbiota in the health benefits of nitrogen-based dietary supplementation [30,31], but its specific role in the ergogenic effects of nitrate remains unclear. Nitrate is actively absorbed by the salivary glands and concentrated in saliva where it is secreted into the oral cavity and partially reduced to nitrite, a process that involves facultative anaerobic bacteria found in the deep clefts on the dorsal surface of the tongue [32]. Select oral bacteria contain specific nitrate reductase enzymes that use nitrate as an alternative electron acceptor to oxygen during respiration.

The gut microbiota also seems to be involved in sports performance. For example, meta-*omics* analysis of the microbiota of elite athletes has identified a performance-enhancing bacterial genus that enhances sports performance via lactate metabolism [33]. In this regard, modulation of the gut microbiota by prebiotics, probiotics, and antibiotics has gained recent attention as being potentially effective for a variety of conditions and also as a sports performance enhancer [34,35]. If the oral and gut microbiota are involved in the nitrite-enhancing functions in sports performance, the possibility of modifying the microbiota to increase its nitrate-to-nitrite conversion, and therefore NO signaling, through diet or the use of supplements, opens a new avenue in the field of sports performance.

The objective of this narrative review is to assess the evidence for a role of the oral and gut microbiota in the enhanced sports performance mediated by dietary nitrate.

## 2. Nitrate Reduction in the Oral Cavity: The Role of Oral Microbiota

The nitrate—nitrite–NO pathway is an alternative system to the classical L-arginine–nitric oxide synthase (NOS) pathway for the generation of NO, supporting and complementing canonical NOS-dependent NO generation [36]. Dietary nitrate is derived mainly from green leafy vegetables such as beetroot, spinach, rocket, kale, celery, fennel and lettuce [37]. Oral bacteria use nitrate and nitrite as final electron acceptors in respiration, aiding the host in the conversion of nitrate to NO. Nitrate is first reduced to nitrite by commensal oral bacteria and, once absorbed into the blood circulation, nitrite is reduced to NO. Nitric oxide synthesis is important in the regulation of vascular tone and blood pressure, by promoting a reduction in vascular tone and blood pressure and improving the bioavailability of NO in different tissues and organs [36,37,38]. Nitric oxide production in the mouth is likely not relevant from a physiological point of view, because even if some oral bacteria do reduce nitrite to NO in saliva, the enzymatic process is slow and nitrite is rapidly extruded through continuous swallowing [39].

**Figure 1:**
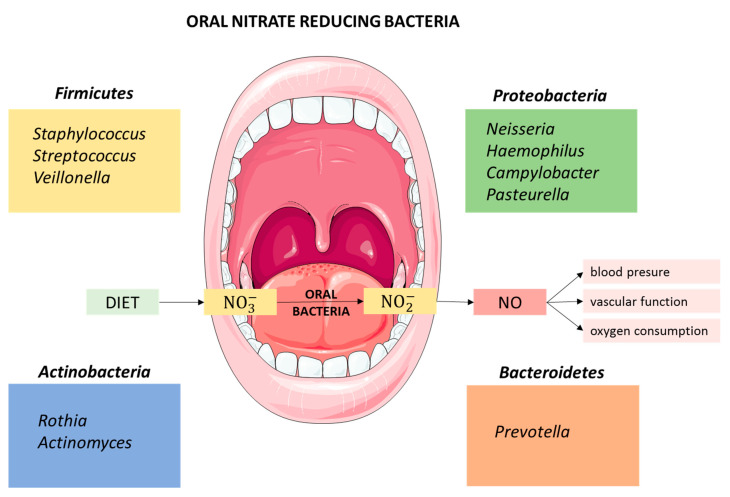
Relevant genera of nitrate-reducing bacteria isolated from the oral cavity.

The oral microbiota is the second most diverse microbial community in the human body, and comprises 50–100 billion bacteria from more than 700 prokaryotic taxa, including a variety of facultative anaerobic bacteria with nitrate reductase activity [40]. Many of these bacteria also contain reductases that enable denitrification of nitrate to, ultimately, nitrogen gas (N_2_) through NO and nitrous oxide (N_2_O) formation [41]. Several bacterial species are strongly implicated in the reduction of nitrate to nitrite. The first identified nitrite-producing bacteria, *Staphylococcus sciuri*, *Staphylococcus intermedius*, *Pasteurella* spp., and *Streptococcus* spp., were isolated from the tongue of adult rats [42]. Recently, bacteria from the genus *Streptococcus*, including *Streptococcus salivarius*, *S. mitis*, *S. bovis* have been reported from human saliva [43]. *Veillonella*, *Actinomyces*, *Rothia*, *Staphylococcus*, *Corynebacterium* and *Propionibacterium* are also important nitrite-producing bacteria, with *Veillonella* being the most abundant group isolated from the tongue of adults, followed by *Actinomyces* [39]. Hyde et al. [44] confirmed *Veillonella* spp. as the most abundant nitrate-reducing genus in the tongue but also detected *Prevotella*, *Neisseria*, and *Haemophilus* at a higher abundance than *Actinomyces* spp. *Veillonella* spp. was also reported as the most abundant oral nitrate reducer in the elderly, indicating that its prevalence in the oral cavity does not change with age [45]. A recent study of oral nitrate-reducing bacteria in healthy individuals confirmed the occurrence of *Prevotella*, *Veillonella* and *Haemophilus*, but also detected *Neisseria* and *Rothia*, with *Prevotella melaninogenica* and *Veillonella dispar* as the most prevalent [46]. A study carried out in infants identified *Prevotella* and *Veillonella* but also *Alloprevotella* and *Leptotrichia* with oral nitrate-reducing activity [47] (Figure 1).

Nitrate supplementation induces significant changes in the oral microbiome. Dietary sodium nitrate was found to increase the abundance of the nitrate-reducing genera *Streptococcus* and *Haemophilus* on the tongue of Wistar rats [48]. Similarly, in patients with hypercholesterolemia, nitrate supplementation (beetroot juice) significantly increased the abundance *of Neisseria flavescens* and *Rothia mucilaginosa* in the oral microbiome [18]. A similar result was reported by Vanthalo et al. [49], who also detected a decrease in the abundance of *Prevotella* and *Veillonella* after nitrate supplementation, which appears to contradict previous studies identifying key oral nitrate-reducing taxa. This could indicate that the increased nitrate availability in the oral cavity may not facilitate the growth of all nitrate-reducing bacteria. Thus, although the abundance of oral nitrate-producers contributes to the regulation of NO bioavailability [46], the total metabolic activity of the nitrate-reducing bacteria might be more important than the individual abundance of each bacterial species involved in this activity [44]. A summary of the studies identifying oral nitrate reducing bacteria is shown in Table 1.

## 3. Gastrointestinal Implications of Dietary Nitrate: A Potential Role of the Gut Microbiota

There is clinical evidence supporting the view that salivary nitrite is important for protection against gastrointestinal diseases. It has been known for many years that antibiotic treatments that inhibit nitrite-producing bacteria in the mouth increase the susceptibility to gastroenteritis [50], and that microbiota dysbiosis can be rescued by dietary nitrate following antibiotic therapy. It has also been reported that the conversion of nitrate to nitrite in the oral cavity “fuels” an important mammalian resistance mechanism against infectious diseases [30], which is controlled by the conversion of nitrite to antimicrobial NO in the acidic stomach. In fact, NO is generated in the human stomach at high concentrations, and its production is dependent on gastric acidity and involves the reduction of salivary-derived nitrite. High concentrations of NO are known to be bactericidal, and this could be a first-line defense against ingested pathogens. Another proposed role for gastric NO is in the regulation of mucosal blood flow and mucus production, two important protective mechanisms for gastric mucosal integrity [51,52]. This is consistent with the observation that the gastric levels of the tight junction proteins occludin and claudin are reduced following microbiota dysbiosis, and are recovered by increasing nitrate intake [53]. There is also evidence that the conversion of nitrate into oxides of nitrogen can prevent the formation of carcinogenic nitrosamines [50] and exert preventive and therapeutic effects in the colonic epithelium in addition to the stomach. This may involve the preservation of an intact adherent mucus layer, which is important to shield the colonic mucosa from bacterial infiltration, and the regulation of epithelial cell restitution [54]. Protective mechanisms could also involve reducing gastrointestinal inflammation. While the mechanisms responsible for the resolution of inflammation in the gut by nitrate metabolism are not well understood, they might involve the balance between the production of reactive nitrogen species (RNS) and the preservation of intestinal integrity [55]. These data, overall, highlight a potential role for gut microbiota in the resolution of inflammation by dietary nitrate. Indeed, a role for RNS derived from nitrate metabolism in maintaining gut microbiota homeostasis has been proposed in mice, with a unique role suggested for the ileum, which prevents bacterial overgrowth. The RNS, possibly peroxynitrite (ONOO-), are highly produced downstream of the reactions of iNOS and the enzyme NADPH oxidase 1 in the ileum of normal healthy mice. The composition and translocation of intestinal bacteria also appear to be regulated by RNS production [53]. iNOS has been described to be upregulated in the colon of patients with inflammatory bowel disease (IBD) [56], and dysbiosis and changes in NO metabolism have been linked to ulcerative colitis, a major form of IBD [57]. However, it is not clear whether dietary nitrate can stimulate the transformation of the bacterial community, for example, through an antimicrobial effect, or whether it is the microbiota and its nitrogen-based metabolic interactions that are responsible for the resolution of dysbiosis. Studies dealing with the effect of dietary nitrate on gut microbiota are summarized in Table 2.

**Figure 2:**
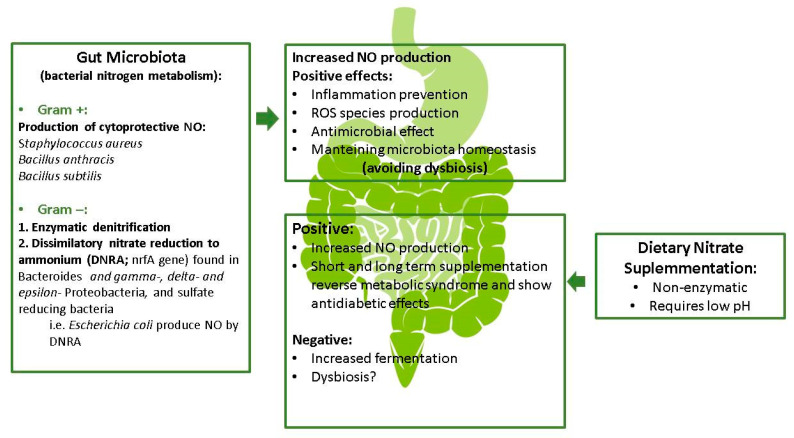
Potential role of nitrogen metabolism in gastrointestinal functions (modified).

Whether an increase in the concentration of nitrate in the intestinal tract (e.g., after consumption of a diet rich in nitrate) produce changes in the microbiota is not clearly understood. While dietary nitrate has a beneficial impact on the gastric and gut mucosa during antibiotherapy [62], this can coincide with an increase in methane production [63], and further studies are needed to clarify these issues and to examine whether gut microbiota dysbiosis occurs after long-term nitrate supplementation as result of enhanced gut fermentation. In mice, long-term dietary nitrite/nitrate deficiency leads to the metabolic syndrome, endothelial dysfunction and cardiovascular death [60], indicating a novel pathogenetic role of the exogenous NO production system in the metabolic syndrome and its vascular complications. This latter study also suggested a causal role of gut microbiota dysbiosis in the development of the metabolic syndrome. By contrast, long-term nitrate supplementation in rodents seems to be effective in preventing the metabolic syndrome [64]. In this line, it has been suggested that the overall observed benefits of nitrate supplementation, and the greater NO production, are found between day 3 and 14 in healthy adults, after which time the supplementation needs to be reviewed [59], both in terms of microbiota and also physiological parameters such as NO. The potential role of nitrogen in the maintenance of the gastrointestinal function is summarized in Figure 2.

The metabolism of nitrate by some Gram-positive bacteria including *Staphylococcus aureus*, *Bacillus anthracis* and *Bacillus subtilis* has been shown to generate NO as a cytoprotective agent against oxidative stress and antibiotics [53,65]. Additionally, a broad range of denitrifying bacteria (mainly Gram-negative) are involved in enzymatic denitrification [66,67], although their role in the gastrointestinal tract is suggested to be minor [68]. The nrfA gene, which encodes a nitrite reductase involved in dissimilatory nitrate reduction to ammonium (DNRA), has been identified in *Bacteroides* species [69] and in gamma-, delta- and epsilon-subclasses of the *Proteobacteria*, and even in sulfate-reducing bacteria [56]. *Escherichia coli*, which is capable of DNRA but not denitrification, has been shown to generate substantial amounts of NO [70]. Whether or not the NO exerts a role in the complex ecosystem of the gut microbiota is not completely known and needs to be explored further.

There is sufficient evidence to indicate a role for the gut microbiota in the metabolism of dietary nitrogen to NO, although it is likely not substantial [56]. The main conversion of dietary nitrate seems to be non-enzymatic and requires an acidic pH, but the acidic conditions could depend on the production of lactic acid by the gut microbiota. While more evidence is needed, it appears that the microbial community creates a specific environment required for the non-enzymatic chemical conversion of nitrate [58].

## 4. Nitrate and the Oral and Gut-Muscle Axis: Potential Ergogenic Effects

Diet-induced changes in the composition of the gut microbiota markedly influence systemic metabolism, fuel availability and exercise capacity. The intake of dietary nitrate might modulate gut microbiota metabolism and contribute to local oxidation-reduction interactions, with a consequent beneficial effect on gut microbiota and health status [66].

Following its reduction by the oral bacteria, the metabolic fate of nitrate once it reaches the blood circulation is relatively unknown. The skeletal muscle has been proposed as a probable site of nitrate buffering, and both animal and human studies have revealed that skeletal muscle can act as a nitrate reservoir that can be drawn on following high intensity exercise [67]. The skeletal muscle appears to have the required molecular machinery for nitrate metabolism, transport, and storage [68]. The presence of a nitrate reservoir should be considered for nitrate supplementation, as it may not be necessary to administer nitrate chronically, but rather on an intermittent basis [28]. Although further studies are necessary to understand the mechanisms involved, it seems that dietary nitrate intake impacts the nitrate stores in skeletal muscle. Accordingly, skeletal muscle could play a key role in the metabolism, transport and storage of nitrate in humans.

Knowledge of the effect that nitrate has on the gut microbiota is very limited. This is in part because it is difficult to isolate its role from that of other compounds provided by dietary vegetable intake. No noteworthy changes in gut microbiota richness have been observed in rodent models of nitrate supplementation [61,62]. As discussed earlier, it is more clear that nitrate is capable of reaching the intestine, both through the digestive system and the bloodstream, where it plays an important protective and anti-inflammatory role in intestinal integrity [54], preserving the habitat of gut microbiota. Some anaerobic bacteria can use nitrate as a nutrient, and also as a final electron acceptor during respiration [71]. Although NOS-like enzymes have been identified in certain enteric bacteria, the vast majority of luminal NO is produced by anaerobic bacteria via the reductive metabolism of nitrate and nitrite [72]. However, the NO produced by this group of bacteria could act as an extra supply for NO bioavailability in skeletal muscle, which might be linked to the improvement of sports performance.

The practice of exercise is a well-known and important modulator of gut microbiota composition, and a very recent report has linked, for the first time, sports performance to the presence of a bacterial species in the gut microbiota [33]. These findings might be reconciled by the existence of a gut-muscle axis, in which the gut microbiota helps to maintain skeletal muscle mass and physical function [34,73]. The notion of crosstalk between the gut microbiota and skeletal muscle actually emerged from several early studies in animals that reported increases in specific bacterial species that produce short-chain fatty acids (SCFAs). It seems that the promotion of bacteria producing SCFAs could be beneficial for skeletal muscle mass and physical function in humans. Physical activity itself is able to stimulate the production of SCFAs, as can a high fiber diet [74]. The practice of physical exercise, in an appropriate manner, has been associated with higher microbiota diversity, an increased presence of health-promoting bacteria and an augmented production of SCFAs [75,76,77]. SCFAs are produced by colon fermentation of undigested carbohydrates and exert their effects both locally and systemically. They protect the integrity of the intestinal barrier and might also be involved in exercise adaptations by regulating intestinal hormones and metabolism, inducing gluconeogenesis and inhibiting lipogenesis [73]. SCFAs and muscle have a direct relationship through the activation of muscular AMP-activated protein kinase (AMPK) by SCFAs. AMPK is involved in the regulation of mitochondrial biogenesis, cholesterol levels and glucose and lipid muscle metabolism [34]. Moreover, SCFAs are also involved in aerobic energy metabolism due to the ability of butyrate, one of the most important SCFAs, to participate in the Krebs cycle [73].

SCFAs produced by the gut microbiota have recently been directly linked to improved exercise performance. Scheiman et al. showed that the abundance of members of the *Veillonella* genus of the gut microbiota was increased in marathon runners after a marathon [33]. Fascinatingly, the administration of *V. atypica* to mice resulted in an increase in their running time as compared with control mice. The authors hypothesized that systemic lactate produced from muscle during physical activity is able to enter the intestinal lumen and is metabolized to propionate by *Veillonella*. Propionate can be used by skeletal muscle to improve exercise performance and has also been shown to increase the heart rate and the maximum rate of oxygen consumption, and to affect blood pressure. When the authors introduced propionate intrarectally to mice, they observed a similar enhancement in exercise performance to that seen after *V. atypica* administration. Thus, the *Veillonella* genus is involved in performance through the metabolism and production of SCFAs (propionate) [33]. By contrast, the administration of antibiotics to mice has been shown to reduce their endurance capacity, as measured by treadmill testing, concomitant with a marked reduction of SCFAs in the cecum and plasma and dysbiosis of the gut microbiota [78]. The administration of acetate, but not butyrate (using an osmotic pump inserted subcutaneously), restored the endurance capacity and, consequentially, acetate could be an energy substrate for endurance exercise [78].

Of note, the same genus of *Veillonella* sp., found in the gut microbiota marathon runners [33] is present in the oral microbiota and is one of the main, if not the main, bacteria involved in the conversion of nitrate to nitrite. This genus thus seems to be strongly linked to sport performance, for its participation in the conversion of nitrate in the oral cavity and for its activity in the intestine as a producer of propionate from lactate generated during physical activity [21].

## 5. Conclusions

In this review, we have discussed the oral and gut bacteria involved in the reduction of dietary nitrate to nitrite and its subsequent conversion to NO, as well as the influence of nitrate on oral and gut microbial populations. The composition of the oral microbiota is modified by the intake of nitrate, favoring (although not all) those bacteria that reduce nitrate. However, the overall effect of the total nitrate-reductase activity in the mouth is unknown. The metabolism of nitrate and its derived compounds plays an important role in the homeostasis of the gastrointestinal system, including the microbiota, but there is a paucity of information on the effect of nitrate intake on the composition of the gut microbiota. Recent studies indicate that certain gut bacteria can enhance exercise performance through their metabolism, underscoring the existence of a microbiota-muscle axis with repercussions for sports performance. The fact that bacterial populations are modifiable with mouthwashes, in the case of oral microbiota, or through the use of probiotics or prebiotics, opens a door to enhance exercise performance through supplementation with nitrate-rich foods. Nevertheless, more research is needed to understand the relationships between nitrate intake and the oral and intestinal microbiota, and the possible storage of nitrate in muscle, which will ultimately lead to a better understanding of the global impact of these factors on sports performance.

## Figures and Tables

**Table 1:** Summary of studies identifying oral nitrate-reducing bacteria with or without nitrate supplementation.

Model	Participants	Age	Nitrate Supple- Mentation	Oral Nitrate-Reducing Bacteria	Reference
Human	hypercholesterolemic n = 69	18–80	Yes (beetroot juice)	*Neisseria*, *Rothia*	[18]
Human	Females (n = 4) Males (n = 6)	29	No	*Veillonella*, *Actinomyces*, *Rothia* and *Staphylococcus*	[39]
Rat	Sprague- Dawley males (n = 5)	adults	No	*Staphylococcus*, *Staphylococcus*, *Pasteurella* spp. and *Streptococcus* spp.	[42]
Human	Females	25–30	No	*Streptococcus*	[43]
Human	n = 6	>18	No	*Veillonella*, *Prevotella*, *Neisseria*, and *Haemophilus*, *Actinomyces*	[44]
Human	Females (n = 12) Males (n = 12)	70.3 ± 6.0	No	*Veillonella*	[45]
Human	n = 25	27 ± 7	No	*Prevotella*, *Veillonella*, *Haemophilus*, *Neisseria*, *Rothia*	[46]
Human	n = 240	2–12 months	No	*Prevotella*, *Veillonella*, *Alloprevotella* and *Leptotrichia*	[47]
Rat	Wistar rats (n = 8)	7 weeks	Yes (NaNO_3_)	*Streptococcus* and *Haemophilus*	[48]
Human	Females (n = 6) Males (n = 3)	70–79	Yes (beetroot juice)	*Rothia*, *Neisseria*	[49]
	Females (n = 5) Males (n = 4)	18–22			

NaNO_3_: sodium nitrate.

**Table 2:** Evidence on the effect of dietary nitrate-based products on the gut microbiota.

Objective	Type of Study and Model	Observed Changes in the Metabolism of the Gut Microbiota	Reference
To test whether the formation of N-nitroso compounds is afforded by the gut microbiota	Pig cecum model	Following incubation with nitrate, the formation of NMOR, NPYR, NMU, and NEU * was detectable, with the microbiota being responsible for the reduction of nitrate to nitrite. After incubation of nitrite, a chemical formation of N-nitroso compounds was observed	[1]
To investigate whether dietary nitrate rescues gastrointestinal physiology during dysbiosis	Wistar rats were maintained in the animal facilities for 7 days during which, in addition to food, they had access to (1) water, (2) an antibiotic cocktail (neomycin, bacitracin, imipenem), (3) antibiotic cocktail plus 10 mM sodium nitrate, (4) sodium nitrate.	Nitrate prevented body weight loss under dysbiosis	[30]
To investigate whether dietary compounds can stimulate NO production	Representative cultures of human gut microbiota	Dietary compounds and the microbial community composition determine the conditions in the colon and hence the chemical production of NO. NO is formed by the reduction of nitrite with hydrogen sulfide, both products of microbial metabolism in the intestinal tract. Only small fractions of NO are released from nitrite, yet these amounts may impact the metabolism of colonocytes	[56]
To investigate whether bacterial nitrate reduction to ammonia, as well as the related NO formation in the gut, could be an important aspect of the overall mammalian nitrate/nitrite/NO metabolism and is yet another way in which the microbiota links diet and health	In vivo bacterial growth cultures	When supplied with exogenous nitrite, *L*. *rhamnosus*, *L. acidophilus* and *B. longum infantis* produce NO independent of added nitrate. Bacterial production of lactic acid causes medium acidification that, in turn, generates NO by non-enzymatic nitrite reduction	[58]
To investigate if changes in the intestinal microbiota induced by a nitrate-rich juice diet play an important role in its health benefits.	Twenty healthy adults consumed only vegetable/fruit juices (rich in nitrate) for 3 days followed by 14 days of customary diet	Between day 4 and 17 there were significant decreases in weight and body mass index Between day 4 and 17, the proportion of Firmicutes and Proteobacteria in stool was significantly decreased whereas Bacteroidetes and Cyanobacteria was increased An increase in NO in plasma was observed on day 4 A 3-day juice (rich in nitrate)-based diet changed the intestinal microbiota associated with weight loss, increased the vasodilator NO, and decreased lipid oxidation	[59]
To test if long-term dietary nitrite/nitrate deficiency induces the metabolic syndrome in mice	Experiments were performed in 6-week-old male C57BL/6J mice	Long-term dietary nitrite/nitrate deficiency gave rise to the metabolic syndrome, endothelial dysfunction and cardiovascular death in mice, indicating a novel pathogenic role of the exogenous NO production system in the metabolic syndrome and its vascular complications	[60]
To investigate the ability of dietary nitrate to improve NO bioavailability and reduce bone turnover and loss in ovariectomized (OVX) rats.	Six-month-old Sprague Dawley rats [30 OVX and 10 sham-operated (sham)] were randomized into three groups: (i) vehicle (water) control, (ii) low-dose nitrate (0.1 mmol nitrate/kg bw/day), or (iii) high-dose nitrate (1.0 mmol nitrate/kg bw/day) for three weeks	OVX (but not dietary nitrate) affects the fecal microbiome and the gut microbiome is associated with bone mass	[61]
To test dietary nitrate as a key component of functional foods with beneficial impact on gastric mucosal integrity during antibiotic therapy	Male Wistar rats followed 4 different treatments: (1) antibiotic cocktail (neomycin, bacitracin and imipenem), (2) antibiotic cocktail + sodium nitrate, (3) sodium nitrate, and (4) regular drinking water.	Dietary nitrate may be envisaged as a key component of functional foods with beneficial impact on gastric mucosal integrity during antibiotic therapy, but further studies are needed to better ascertain as to whether it modulates intestinal microbiota in terms of taxonomic and functional levels	[62]
To evaluate the effects of using encapsulated nitrate product (ENP) on ruminal fermentation	Ruminal fermentation and methane production in vitro using a semi-automatic gas production technique	ENP increases methane production	[63]

* N-nitrosomorpholine (NMOR) and N-nitrosopyrrolidine (NPYR), and the N-nitrosamides, N-nitrosomethylurea (NMU) and N-nitrosoethylurea (NEU).
